# Serum miRNA Changes Associated with Grape Pomace Supplementation in Dairy Sheep

**DOI:** 10.3390/ani16152305

**Published:** 2026-07-25

**Authors:** Gabriella Guelfi, Cecilia Dall’Aglio, Francesca Mercati, Camilla Capaccia, Nicole Maculet, Vicente Francisco Ratto, Francesco Ciancabilla, Margherita Maranesi

**Affiliations:** Department of Veterinary Medicine, University of Perugia, 06126 Perugia, Italy; cecilia.dallaglio@unipg.it (C.D.); francesca.mercati@unipg.it (F.M.); camilla.capaccia@dottorandi.unipg.it (C.C.); vicentefrancisco.rattovalderrama@dottorandi.unipg.it (V.F.R.); francesco.ciancabilla@studenti.unipg.it (F.C.); margherita.maranesi@unipg.it (M.M.)

**Keywords:** grape pomace, circulating microRNAs, nutritional epigenetics, metabolic adaptation, dairy sheep

## Abstract

Grape pomace (GP) is being explored as a sustainable feed ingredient for ruminants due to its fiber and polyphenol content; however, its systemic effects under farming conditions remain unclear. This study tested whether 5% dried grape pomace (GP5) modulates serum circulating miRNAs (cmiRNAs) in dairy sheep. Twelve Sarda ewes were enrolled in a 30-day trial and assigned to a control (CTRL) or GP5 diet. Small RNA sequencing was initially performed in GP5 animals (paired T30 vs. T0 comparison); after quality filtering, five paired animals were included in the sequencing analyses. The differentially expressed (DE) cmiRNAs were validated by RT-qPCR in GP5 and CTRL groups. RT-qPCR confirmed the NGS-derived miRNA signature, identifying miR-143, miR-382-5p, miR-409-3p, and miR-194 as significantly downregulated (*p* < 0.05) in GP5 animals. Ingenuity Pathway Analysis (IPA) showed that DE miRNAs target shared hubs, with PPAR signaling among the most enriched pathways (BH-adjusted *p* = 1.35 × 10^−9^), predicted to be inhibited (Z = −2.646). Cholecystokinin/gastrin-mediated and S100 signaling were also enriched (BH-adjusted *p* = 7.08 × 10^−11^ and 3.21 × 10^−8^) and predicted to be activated (Z = 2.828 and 3.162). Overall, GP5 supplementation was associated with a selective cmiRNA signature consistent with targeted post-transcriptional regulation.

## Simple Summary

Grape pomace, a by-product of the wine industry, is increasingly used as a sustainable feed ingredient in ruminant nutrition. However, its effects at the molecular level are not fully understood. In this study, we investigated whether a diet supplemented with 5% grape pomace influences circulating microRNAs in dairy sheep. Small RNA sequencing identified a specific set of circulating microRNAs that were consistently reduced after supplementation and were associated, based on Ingenuity Pathway Analysis, with metabolic and inflammatory regulatory pathways. These findings suggest that grape pomace may act as a natural nutritional modulator, supporting metabolic adaptation while preserving systemic health in dairy sheep.

## 1. Introduction

Sustainable livestock production increasingly depends on the capacity to valorize agro-industrial by-products as biologically active feed resources capable of supporting metabolic resilience without compromising animal health. Among these, grape pomace (GP), a major residue of the winemaking industry, has emerged as a promising functional ingredient for ruminants due to its high fiber and polyphenol content and its wide availability [1,2,3,4]. Beyond its nutritional value, GP represents a strategic component of circular-economy models, enabling the sustainable reuse of viticultural by-products while supporting livestock productivity [1,4]. Recent studies have further highlighted grape pomace as a sustainable functional feed ingredient capable of influencing not only productive performance but also host metabolic regulation and gut microbial ecology [5,6,7].

From a physiological perspective, GP supplementation can modulate rumen fermentation dynamics by influencing microbial interactions and shifting the balance between acetate and propionate production [1,2,3]. Because propionate is the principal gluconeogenic substrate in ruminants, such alterations may directly impact hepatic glucose synthesis and systemic energy homeostasis [1,2,3]. Moreover, dietary polyphenols have been reported to influence oxidative and inflammatory pathways, although the magnitude and direction of these responses vary depending on species, inclusion level, and production system [4]. In dairy ewes, low inclusion rates (such as 5%) are currently being investigated as a practical strategy to support metabolic stability during periods of increased physiological demand [8].

Beyond these physiological effects, a critical question concerns how dietary bioactives translate into coordinated regulatory adaptation at the molecular level. Increasing evidence indicates that dietary phytochemicals modulate gene regulatory networks, including microRNA-mediated post-transcriptional regulation, thereby contributing to systemic metabolic adaptation and providing a mechanistic basis for the use of circulating miRNAs as nutritional biomarkers [9,10].

Circulating microRNAs (cmiRNAs) are small non-coding RNAs released into the bloodstream from multiple tissues, where they exhibit high stability and reflect coordinated systemic adaptation [11,12]. In ruminants, shifts in energy balance, inflammatory tone, or nutritional stress are accompanied by specific changes in cmiRNA profiles, often preceding measurable variations in conventional biochemical parameters [11,13]. Several cmiRNAs have been associated with pathways involved in glucose metabolism, lipid handling, oxidative balance, and immune activation, including NF-κB, AMPK, and PPAR signaling axes [11,14,15,16]. Within the framework of nutritional epigenetics, dietary compounds may therefore exert regulatory effects through selective modulation of miRNA-mediated post-transcriptional control [14].

Despite growing interest in GP as a functional feed ingredient, its impact on cmiRNA signatures in dairy ewes under practical farming conditions remains largely unexplored [3,4]. Recent findings from our group demonstrated that 5% GP supplementation in Sarda dairy ewes is metabolically safe and associated with measurable systemic and productive adaptations [8]. However, whether these physiological adjustments are underpinned by a coherent post-transcriptional regulatory signature remains unresolved. In particular, it remains unclear whether the metabolic adaptations associated with GP supplementation are accompanied by a coordinated reshaping of the cmiRNA landscape, indicative of a regulated molecular response rather than generalized metabolic perturbation.

To address this gap, the present study aimed to define, for the first time in vivo, the cmiRNA regulatory signature associated with 5% GP supplementation in dairy ewes. By integrating small-RNA next-generation sequencing with RT-qPCR validation and pathway-level bioinformatic reconstruction, we sought to identify nutritionally responsive cmiRNAs and to determine whether their functional convergence supports the presence of a biologically coherent, system-level regulatory adjustment under practical farming conditions.

## 2. Materials and Methods

### 2.1. Animal Enrollment, Study Design, and Dietary Intervention

**Figure 1:**
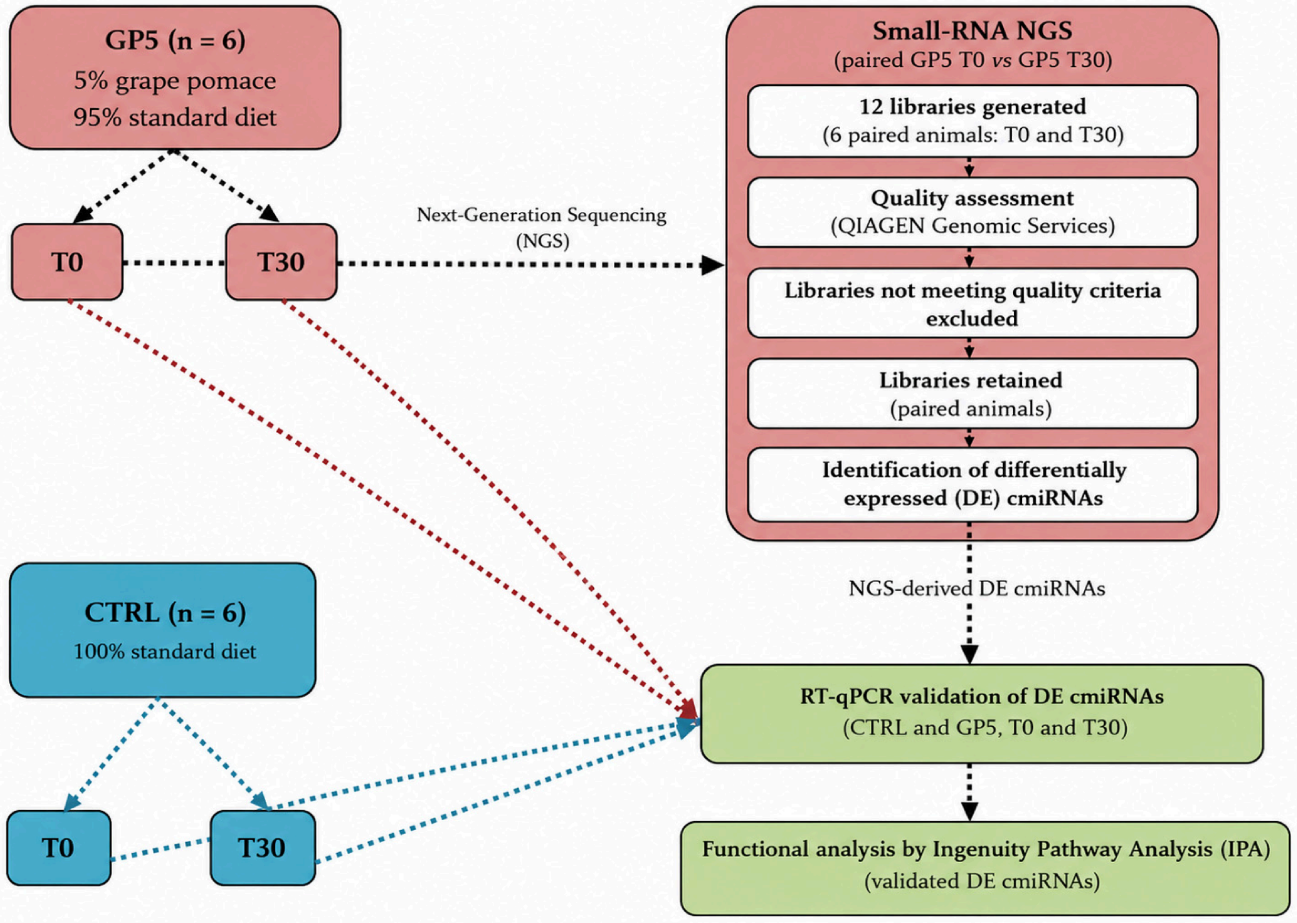
Experimental design and analytical workflow of the study. Twelve Sarda dairy ewes were randomly assigned to two groups: CTRL (blue box, 100% standard diet, *n* = 6) and GP5 (purple box, 95% standard diet + 5% dried grape pomace, *n* = 6). Blood samples were collected at baseline (T0) and after 30 days of dietary administration (T30). Small-RNA sequencing was performed exclusively on GP5 samples using a paired design (GP5 T0 vs. GP5 T30). Twelve sequencing libraries were initially generated from six paired GP5 animals. Following quality assessment by QIAGEN Genomic Services, libraries that did not meet the provider’s predefined quality control criteria were excluded prior to downstream bioinformatic analyses. Differentially expressed (DE) cmiRNAs identified by sequencing were subsequently validated by RT-qPCR using serum samples collected from both GP5 and CTRL animals at T0 and T30. Finally, the RT-qPCR-validated DE cmiRNAs were subjected to Ingenuity Pathway Analysis (IPA) to investigate their associated biological pathways.

The study was conducted on 12 Sarda dairy ewes, a Mediterranean breed widely used in Central Italy for milk production. All animals were adult, clinically healthy, and homogeneous in age, body condition, and management, with an average age of 3–5 years and a mean body weight of 55 ± 5 kg. The experimental protocol was approved by the Ethics Committee of the University of Perugia (protocol code No. 46269; approval date: 14 February 2023). Ewes were in late lactation and were randomly allocated to two homogeneous groups based on body weight, milk yield, and physiological status: CTRL (*n* = 6), fed a standard pelleted diet; and GP5 (*n* = 6), fed a pelleted diet consisting of 95% basal pelleted diet and 5% dried grape pomace on a dry matter basis. The formulation and proximate chemical composition of the experimental diets are provided in Supplementary Table S2. The overall experimental design, dietary formulation, and animal management procedures followed those previously described for the same animal cohort [8]. Feed was administered twice daily, and animals had free access to clean drinking water. All animals consumed the entire daily ration provided (2.1 kg DM/ewe/day). Accordingly, the estimated grape pomace intake in the GP5 group was approximately 105 g DM/ewe/day. The feeding trial lasted 30 consecutive days, with blood samples collected at baseline (T0) and at the end of the experimental period (T30). Small-RNA sequencing was performed exclusively on GP5 samples (T0 vs. T30, paired design) as an exploratory discovery step to identify candidate cmiRNAs responsive to grape pomace supplementation while minimizing biological variability and analytical costs. Quantitative PCR validation of the DE cmiRNAs identified by sequencing was subsequently conducted in both experimental groups (CTRL and GP5) at T0 and T30 (Figure 1).

### 2.2. GP Drying, Extraction, Polyphenol Characterization, and Pelleted Feed Formulation

The GP used in this study was obtained from the skin fraction of fermented red winemaking by-products derived from local red grape cultivars from Montesperello di Magione, Perugia, Umbria (Italy) after mechanical seed separation. The same GP source and processing procedure were previously described [8]. The skin fraction was dried in a ventilated oven at ≤40 °C until the residual moisture content was below 10%, then coarsely ground and homogenized before incorporation into the experimental diet. After drying, the GP was stored under dry conditions at room temperature until feed preparation. For phytochemical characterization, a representative subsample of the dried GP was subjected to methanolic extraction exclusively for analytical purposes. This extraction was performed solely for phytochemical characterization of the grape pomace extract and was not involved in feed preparation or animal supplementation. The UHPLC–HRMS analysis was used for phytochemical characterization and compound identification. No comprehensive quantitative profiling of the GP extract was performed. Animals received only the native dried GP incorporated into the pelleted diet. UHPLC–HRMS analysis was performed using a Vanquish UHPLC system coupled to an Orbitrap Exploris™ 120 high-resolution mass spectrometer (Thermo Fisher Scientific, Bremen, Germany) equipped with a Phenomenex PS C18 column (100 × 2.1 mm, 1.6 μm). Mass spectra were acquired in negative electrospray ionization mode. Compound identification was based on accurate mass measurements, chromatographic retention time, and MS/MS fragmentation patterns by comparison with the corresponding authentic reference standards. Chromatographic data were processed using Chromeleon™ 7.3.1 software (Thermo Fisher Scientific, Waltham, MA, USA). The analytical extraction procedure has been previously described in detail [8], whereas the UHPLC–HRMS phytochemical characterization of the GP extract used in the present study, including the identified compounds and the base peak chromatogram, is provided in Supplementary Table S1 and Supplementary Figure S1. The GP5 diet was formulated by incorporating 5% dried GP into 95% of the basal CTRL diet on a dry matter basis. The mixture was thoroughly homogenized and pelleted by a commercial feed manufacturer (Azienda Mangimistica Grigi, Pontenuovo di Torgiano, Perugia, Italy) under standard industrial processing conditions. Exact information on conditioning temperature, pelleting duration, pellet diameter, and moisture immediately after pelleting was not recorded or made available by the manufacturer. However, the final pelleted diets had dry matter contents of 86.78% for CTRL and 86.92% for GP5, as determined by proximate analysis (Supplementary Table S2b). Pellets were stored under dry conditions until use. The ingredient formulation and proximate chemical composition of the experimental diets are reported in Supplementary Table S2a,b.

### 2.3. Hematological Analyses

The systemic safety of the GP5 dietary treatment was evaluated by monitoring biochemical parameters. Animals were subjected to routine veterinary monitoring throughout the experimental period to assess general health status and detect any clinical signs of disease. Blood samples collected at T0 and T30 (CTRL and GP5 groups) were analyzed using an automated clinical chemistry analyzer (KeyLab System, Menarini Diagnostics S.r.l., Florence, Italy) following standard laboratory procedures. Hematological variables, including red and white blood cell counts, hemoglobin concentration, hematocrit, and leukocyte differential, were determined using an automated hematology counter (HeCo Vet C, SEAC Radim, Calenzano, Florence, Italy). Serum samples were also assessed for hemolysis as part of the pre-analytical quality control procedure prior to RNA extraction. Hemolysis was initially evaluated by visual inspection for pink discoloration and subsequently by measuring the absorbance of free hemoglobin at 414 nm using a NanoDrop 1000 spectrophotometer (Thermo Fisher Scientific, Waltham, MA, USA).

### 2.4. RNA Extraction, Library Preparation, and cmiRNA Sequencing

Total RNA, including small RNA species, was extracted by the authors from 200 μL of serum using the MiRNeasy Serum/Plasma Advanced Kit (QIAGEN, Hilden, Germany), following the manufacturer’s protocol. RNA was eluted in 14 μL of RNase-free water. Quantification was performed using the Qubit RNA HS Assay Kit (Thermo Fisher Scientific, Waltham, MA, USA), and RNA integrity was assessed with the RNA IQ Assay (Thermo Fisher Scientific, Waltham, MA, USA).

Library preparation and sequencing were carried out by QIAGEN Genomic Services (Hilden, Germany). For each sample, 5 μL of total RNA was used as input for the QIAseq miRNA Library Kit (QIAGEN). Adapter ligation and reverse transcription were performed according to the manufacturer’s protocol, introducing Unique Molecular Indexes (UMIs) during cDNA synthesis to correct amplification bias and enable absolute quantification. Amplified libraries (22 PCR cycles) were purified and evaluated by capillary electrophoresis (TapeStation D1000, Agilent Technologies, Santa Clara, CA, USA) to confirm the expected insert size and absence of adapter dimers. Libraries were quantified by qPCR, normalized, and pooled in equimolar amounts before sequencing. The final pooled library was sequenced on an AVITI platform (Element Biosciences, San Diego, CA, USA) using a 1 × 76 bp configuration with 2 × 10 bp index reads, according to standard QIAGEN protocols. Raw reads were demultiplexed and exported as FASTQ files using the bases2fastq software (version 00.000.000.4.2.4). Twelve libraries were initially generated (six paired GP5 animals at T0 and T30). Following the quality assessment performed by QIAGEN Genomic Services, two libraries did not meet the provider’s quality criteria for downstream bioinformatic analysis and were therefore excluded prior to differential expression analysis. Consequently, all subsequent sequencing analyses were performed on five paired GP5 animals (10 libraries).

### 2.5. Differential Expression Analysis

Raw CPM values generated by QIAGEN Genomic Services were provided in the Supplementary NGS data.xlsx. Lowly expressed miRNAs were filtered by retaining only those with CPM > 1 in ≥50% of samples, thereby minimizing low-coverage artefacts. The resulting filtered dataset (“Filtered_CPM_matrix.xlsx”) was used for both endogenous reference (ER) selection and differential expression (DE) analysis. For DE analysis, demultiplexed FASTQ files were trimmed, UMI-deduplicated, aligned to the Ovis aries reference genome (Ensembl release 111), and annotated against miRBase v22.1. Count data were imported into R 4.5.1 and analyzed using the limma-voom pipeline (edgeR + limma packages). Library sizes were normalized using the trimmed mean of M values (TMM) method. A paired linear model was implemented (~Animal + Time) to account for within-animal variation (GP5 T0 vs. T30). Empirical Bayes moderation was applied, and miRNAs with FDR < 0.05 were considered significantly differentially expressed. Linear fold changes were calculated as 2^(log_2_FC). Differential expression results were independently validated using DESeq2, yielding an identical four-miRNA signature, thereby confirming the robustness of the detected modulation. All bioinformatic analyses described below were performed using the final dataset of ten libraries (five paired GP5 animals).

### 2.6. Endogenous Reference cmiRNA Selection and Stability Validation

Candidate ER cmiRNAs were selected through a combined data-driven and literature-supported approach. Initially, the filtered NGS dataset, comprising 10 libraries from five paired GP5 animals sampled at T0 and T30, was screened to identify miRNAs with adequate abundance (CPM > 1 in ≥50% of samples) and relatively low variability, based on mean expression, standard deviation (SD), and coefficient of variation (CV%). Previous studies have evaluated miR-16, miR-191, and miR-103 as candidate or validated reference miRNAs in different human biological matrices [17,18,19]. Based on these criteria, oar-miR-191-5p, oar-miR-16b-5p, and oar-miR-103a-3p were selected as candidate ER cmiRNAs. Their expression stability was subsequently evaluated using RT-qPCR data from all experimental samples, including CTRL and GP5 animals at T0 and T30, using RefFinder [20], which integrates the ΔCt method [21], BestKeeper [22], NormFinder [23], and GeNorm [24].

### 2.7. qPCR Validation of cmiRNAs

QPCR validation of selected DE cmiRNAs was performed on CTRL (T0 and T30) and GP5 (T0 and T30) using the same serum samples (and corresponding RNA extracts) employed for small-RNA sequencing using the miRCURY LNA miRNA PCR System (QIAGEN, Hilden, Germany). RT-qPCR validation included all twelve experimental animals (CTRL, *n* = 6; GP5, *n* = 6), independently of the subset retained for small-RNA sequencing following quality assessment. Total RNA was reverse transcribed with the miRCURY LNA RT Kit (QIAGEN) following the manufacturer’s instructions. Each 10 μL reaction contained 2 μL of RNA, 2 μL of 5 × RT Reaction Buffer, 1 μL of Enzyme Mix, and 5 μL of nuclease-free water. Reverse transcription was carried out at 42 °C for 60 min, followed by enzyme inactivation at 95 °C for 5 min. The resulting cDNA was diluted 1:10 and amplified using miRCURY LNA SYBR Green PCR assays (QIAGEN) specific for each target cmiRNA (Table 1).

Each qPCR reaction (10 μL) contained 5 μL of SYBR Green Master Mix, 1 μL of LNA primer assay, 1 μL of diluted cDNA, and 3 μL of RNase-free water. Amplifications were performed in triplicate on a StepOnePlus Real-Time PCR System (Applied Biosystems, Foster City, CA, USA) with the following cycling conditions: initial activation at 95 °C for 2 min, followed by 40 cycles of 95 °C for 10 s and 56 °C for 60 s. Melting curve analysis was used to verify amplicon specificity. For data normalization, oar-miR-191-5p, oar-miR-16b-5p, and oar-miR-103a-3p were selected as ER cmiRNAs based on their stable expression across all samples. UniSp6 was included as an exogenous spike-in control to monitor reverse transcription efficiency [25]. Quantification cycle (Cq) values were obtained using StepOne software v2.3 with a fixed threshold of 0.2. Only amplifications with a Ct ≤ 35 and a single melting peak were retained for analysis. Relative expression levels were calculated by the 2^−ΔCq method. ΔCq was calculated as the difference between the Cq value of each target cmiRNA and the selected endogenous reference [26].

### 2.8. Statistical Analysis

Small-RNA sequencing was performed on the subset of GP5 samples retained after quality assessment, using a paired comparison (T30 vs. T0 within the same animals). Differential expression of cmiRNAs was assessed using the limma-voom pipeline, as detailed in the Section 2.5. cmiRNAs with a false discovery rate (FDR) < 0.05 were considered significantly differentially expressed, and linear fold changes were calculated. The Empirical DGE module of CLC Genomics Workbench was used exclusively for preliminary inspection of sequencing quality and expression data and was not used for the differential expression results reported in this manuscript. For the RT-qPCR validation dataset and phenotypic variables, an overall two-way repeated-measures ANOVA (Group × Time) was first performed, followed, where appropriate, by planned pairwise comparisons (T30 vs. T0 within each group and CTRL vs. GP5 at each time point). Data normality and homogeneity of variances were assessed using the Shapiro–Wilk and Levene tests, respectively. Planned pairwise comparisons were performed using Bonferroni’s multiple-comparison test. When the assumptions for parametric analysis were not met, the corresponding non-parametric tests (Wilcoxon signed-rank test for paired comparisons and Mann–Whitney U test for unpaired comparisons) were applied. Technical triplicates were averaged prior to statistical analysis after excluding wells with a coefficient of variation (CV) > 2% or multimodal melting curves. Results are presented as mean ± SEM, and statistical significance was set at *p* < 0.05. All statistical analyses were performed using GraphPad Prism version 10.5 (GraphPad Software, La Jolla, CA, USA). For each validated cmiRNA, the Group effect, Time effect, Group × Time interaction, F-values, degrees of freedom, and corresponding *p*-values were determined.

Ingenuity Pathway Analysis (IPA, QIAGEN) was used as a hypothesis-generating approach to explore potential pathway-level associations of the RT-qPCR-validated cmiRNAs associated with GP5 supplementation. The analysis was performed by mapping ovine miRNAs to their human orthologs and considering only experimentally validated and high-confidence predicted targets. Canonical pathway and network enrichment analyses were conducted to identify biological processes potentially associated with the validated cmiRNA signature. IPA results were interpreted descriptively and used for hypothesis generation without inferring direct causal or mechanistic relationships.

## 3. Results

### 3.1. Systemic Health and Hematological Profile

Animals showed no clinical signs of ruminal dysfunction or systemic illness during the experimental period. No cases of acidosis-related manifestations (decreased feed intake, altered rumination, diarrhea, discomfort) were observed, and no treatments or veterinary interventions were required. Hematological parameters further confirmed the absence of adverse effects associated with GP dietary inclusion. No significant differences were observed between T0 and T30 within either CTRL or GP5 groups (*p* > 0.05). White blood cells (WBC), lymphocytes (LYM), monocytes (MONO), and granulocytes (GRAN) remained stable over time. Similarly, erythrocyte indices, including hemoglobin (HGB), hematocrit (HCT), red blood cells (RBC), mean corpuscular volume (MCV), mean corpuscular hemoglobin (MCH), and mean corpuscular hemoglobin concentration (MCHC), did not show significant variations. Platelet count (PLT) and mean platelet volume (MPV) also remained unchanged. All values were within or close to physiological reference ranges.

### 3.2. Sequencing Performance and QC Metrics

Quality assessment of the sequencing reads was first performed by QIAGEN Genomic Services. Twelve libraries were initially generated from six paired GP5 animals sampled at T0 and T30. Following quality assessment, two libraries did not meet the provider’s quality criteria and were excluded from downstream bioinformatic analyses. Consequently, all sequencing analyses were performed on the remaining ten libraries, corresponding to five paired GP5 animals. The overall quality of the reads was excellent, with more than 90% showing PHRED scores between 40 and 50 and no ambiguous bases detected. GC content was stable (mean 54% ± 2.5%), and sequence duplication levels were low (corresponding to approximately 90% unique reads). No technical problems or outliers were identified, confirming that the remaining ten libraries fulfilled the quality requirements for downstream bioinformatic analysis.

### 3.3. NGS-Based Differential Expression Analysis of cmiRNAs

Following library quality assessment, differential expression analysis was performed on the five paired GP5 animals retained for sequencing. The paired comparison (GP5 T30 vs. T0) identified four significantly differentially expressed cmiRNAs (FDR < 0.05; limma-voom pipeline), which were subsequently selected for RT-qPCR validation. All four cmiRNAs, oar-miR-143, oar-miR-409-3p, oar-miR-382-5p, and oar-miR-194, displayed a consistent pattern of downregulation at T30 compared to T0 (raw *p*-values ranging from 0.00027 to 0.00094). The magnitude of modulation ranged from approximately 1.9- to 2.5-fold reduction in expression, with oar-miR-409-3p showing the greatest decrease (~2.50-fold lower expression at T30; log_2_FC = −1.32). This was followed by oar-miR-143 (~2.20-fold; log_2_FC = −1.14), oar-miR-194 (~1.91-fold; log_2_FC = −0.94), and oar-miR-382-5p (~1.90-fold; log_2_FC = −0.92). The same four-miRNA signature was independently reproduced using DESeq2, yielding concordant direction and magnitude of change, thereby supporting the robustness of the identified modulation across analytical frameworks. A comprehensive summary of log_2_ fold changes, linear fold-changes, average log_2_ expression levels (AveExpr), raw *p*-values, FDR-adjusted *p*-values, and expression trends is provided in Table 2.

### 3.4. ER cmiRNAs Selection

Following their preliminary selection from the filtered NGS dataset (Supplementary NGS data; candidate ER cmiRNAs are highlighted in yellow), the expression stability of oar-miR-191-5p, oar-miR-16b-5p, and oar-miR-103a-3p was evaluated using RT-qPCR Cq data from CTRL and GP5 samples at T0 and T30. RefFinder integrated the ΔCt method, BestKeeper, NormFinder, and GeNorm rankings. Oar-miR-16b-5p was ranked as the most stable individual candidate by the ΔCt method, BestKeeper, NormFinder, and the comprehensive RefFinder analysis [27]. GeNorm identified the oar-miR-16b-5p/cfa-miR-191 pair as the most stable combination, whereas oar-miR-103a-3p showed the lowest stability. Based on these results, oar-miR-16b-5p was selected as the endogenous reference cmiRNA for RT-qPCR normalization (Table 3).

### 3.5. qPCR Validation of DE cmiRNAs

RT-qPCR assay performance and quality control were first evaluated to ensure analytical reliability. Following confirmation of serum sample integrity and absence of significant hemolysis (A414 values ranged from 0.068 to 0.120, all below the predefined acceptance threshold of 0.2), RNA isolation and reverse transcription quality were further verified using the spike-ins UniSp2 and UniSp4 (RNA isolation controls) and UniSp6 (reverse transcription control). All samples showed consistent Cq values within the expected range (Cq ≤ 35), and no reactions were excluded from downstream analysis. Relative expression of the four DE cmiRNAs (miR-143-3p, miR-409-3p, miR-382-5p, and miR-194-5p) was normalized to the most stable ER, miR-16b-5p, as identified in Section 3.4, and quantified using the 2^−ΔCq method. RT-qPCR validation showed no significant differences among CTRL T0, CTRL T30, and GP5 T0 samples for any of the analyzed cmiRNAs (*p* > 0.05), confirming baseline comparability across groups and temporal stability in CTRL animals.

Following the overall two-way repeated-measures ANOVA, planned pairwise comparisons were performed to further investigate temporal changes within each group. Significant Group × Time interactions were observed for all four validated cmiRNAs, indicating that the temporal changes differed significantly between the CTRL and GP5 groups. The results of the overall ANOVA together with the planned pairwise comparisons are summarized in Table 4.

**Figure 2:**
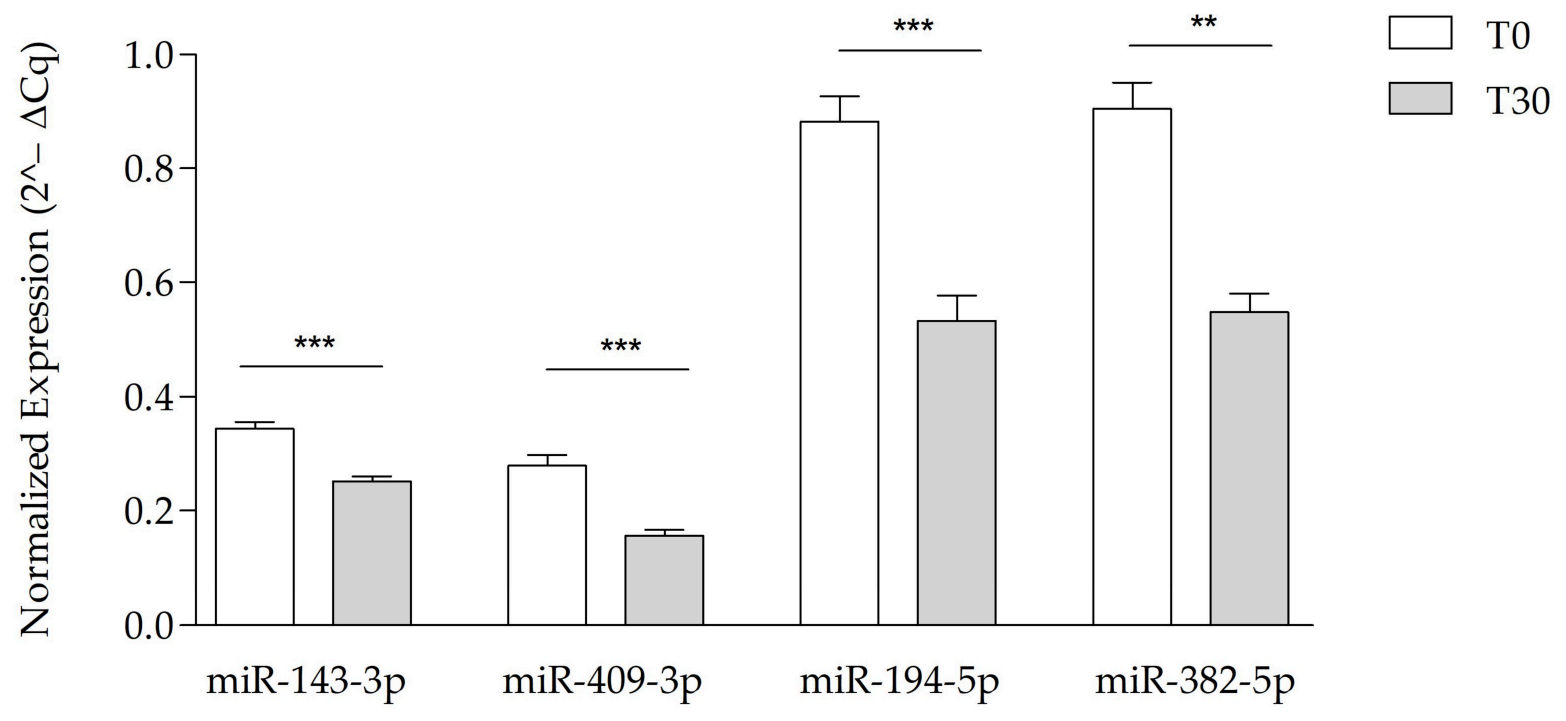
RT-qPCR validation of differentially expressed cmiRNAs in the GP5 group. Normalized expression values (2^−ΔCq) of miR-143-3p, miR-409-3p, miR-194-5p, and miR-382-5p at baseline (T0) and after 30 days of grape pomace supplementation (T30). Bars represent mean ± SEM (*n* = 6). Asterisks indicate significant differences between T0 and T30 within the GP5 group (*** *p* < 0.001; ** *p* < 0.01).

Normalized expression data (2^−ΔCq) further confirmed the coordinated downregulation of all validated cmiRNAs in the GP5 group after 30 days of dietary supplementation. Compared with baseline (T0), all four validated cmiRNAs showed significantly lower expression at T30, confirming the marked reduction in expression following GP5 supplementation (Figure 2).

### 3.6. IPA of Downregulated microRNAs

**Figure 3:**
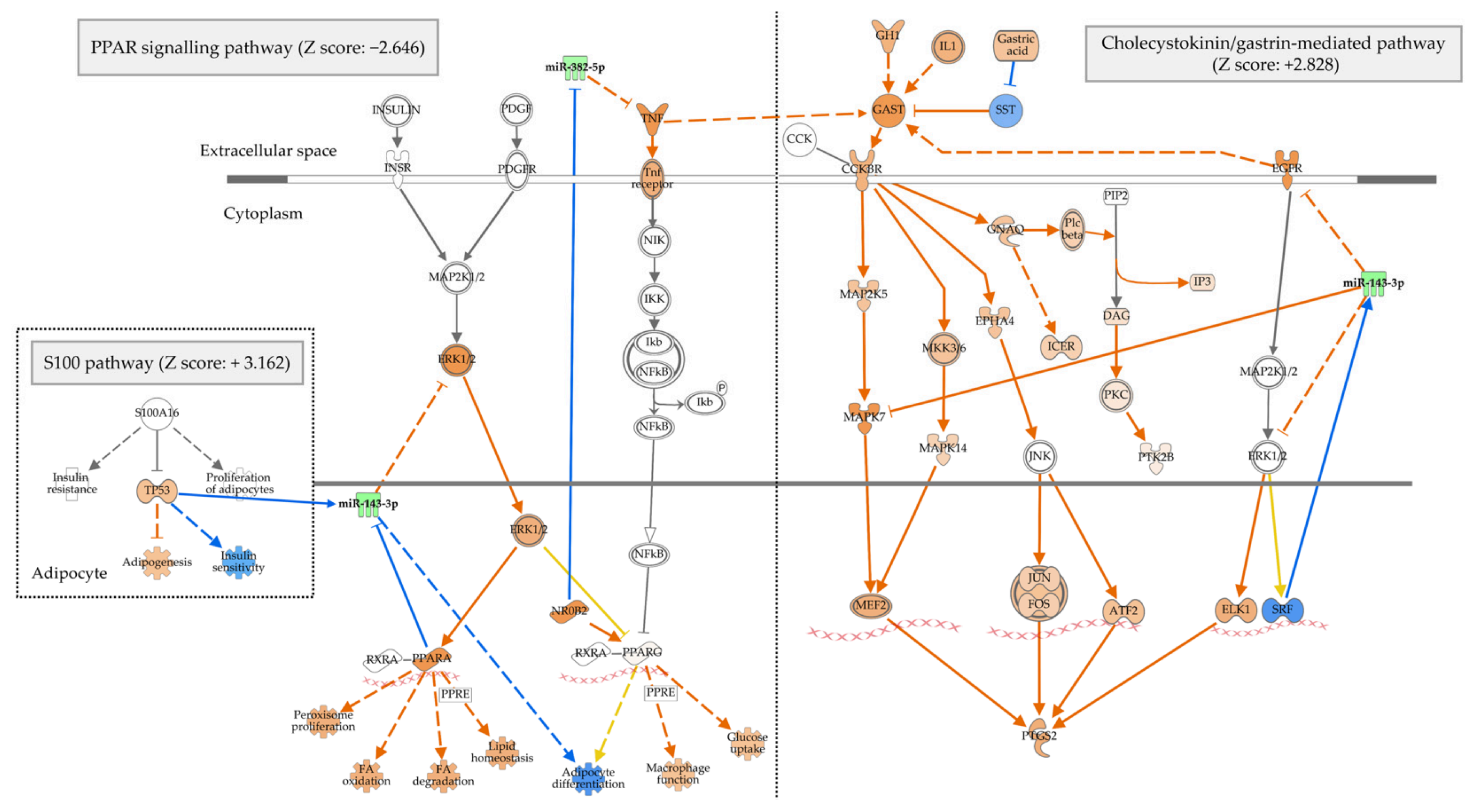
Integrated IPA network of PPAR, cholecystokinin/gastrin, and S100 signaling pathways. The figure shows an IPA-generated network integrating the PPAR signaling pathway (**left**), the cholecystokinin/gastrin-mediated signaling pathway (**right**), and a focused S100 signaling module (**bottom left**). The downregulated miRNAs miR-143-3p and miR-382-5p are shown as green nodes and emerge as the main miRNA regulators represented in the integrated network. Orange nodes indicate predicted activation, whereas blue nodes indicate predicted inhibition; color intensity reflects the strength of the predicted state. Solid lines denote direct interactions, whereas dashed lines indicate indirect relationships. Edge colors indicate the predicted effect of the interaction: orange, activation; blue, inhibition; yellow, inconsistency with the predicted downstream state; gray, effect not predicted. Within the reconstructed network, miR-143-3p and miR-382-5p are associated with regulatory nodes associated with PPARα/γ signaling, MAPK/ERK-related modules, and S100-associated signaling components. In the PPAR module, upstream cues mediated by PDGF/PDGFR and INSR converge on the SHC1–GRB2–SOS–RAS–RAF1–MAP2K1/2 cascade, linking growth factor and insulin-responsive signaling to nuclear metabolic regulators. In the cholecystokinin/gastrin module, signaling initiated by GAST/CCK through CCKBR and EGFR connects to intracellular Ca^2+^-, PKC-, and MAPK-related pathways and to downstream transcription factors including AP-1-associated nodes. The S100 module includes S100A16 and TP53 and is positioned in relation to adipocyte-associated metabolic and stress-responsive functions. Overall, the integrated network provides a graphical representation of the pathway-level convergence identified by IPA and supports the interpretation of a coordinated regulatory framework underlying the GP5-associated cmiRNA signature.

IPA was employed as a hypothesis-generating approach to explore potential pathway-level associations of the four cmiRNAs significantly downregulated following GP5 supplementation. Ovine miRNAs were mapped to their annotated human homologs to enable network and canonical pathway analyses. Among the modulated cmiRNAs, miR-143-3p and miR-382-5p contributed most prominently to canonical pathway enrichment with computable activation Z-scores, whereas miR-409-3p and miR-194 were primarily represented within the broader miRNA–target interaction network, consistent with differences in target annotation density. Overall, IPA indicated that validated, high-confidence predicted targets for the four cmiRNAs converged on a limited set of shared regulatory nodes, supporting the presence of a coordinated, system-level signature rather than isolated molecular events. PPAR signaling emerged as one of the most significantly enriched pathways (*p* = 6.53 × 10^−11^; BH-adjusted *p* = 1.35 × 10^−9^), with a negative activation Z-score (Z = −2.646), indicating predicted attenuation at the pathway level. Within the reconstructed network, miR-143-3p and miR-382-5p were associated with regulatory nodes involved in PPARα/γ signaling and downstream functions, including lipid handling, fatty acid oxidation, adipocyte differentiation, and glucose metabolism. Conversely, the Cholecystokinin/gastrin-mediated signaling was also strongly enriched (*p* = 2.36 × 10^−12^; BH-adjusted *p* = 7.08 × 10^−11^) but showed a positive activation Z-score (Z = +2.828), and network integration positioned the same cmiRNAs within MAPK/ERK- and AP-1-associated modules, with PTGS2 (COX-2) identified as a relevant downstream node. The S100 signaling pathway was likewise significantly enriched (*p* = 3.69 × 10^−8^; adjusted *p* = 3.21 × 10^−8^) with a positive activation score (Z = + 3.162), and network reconstruction suggested associations between GP5-modulated cmiRNAs and S100-related signaling nodes, including S100A16, within adipocyte-related metabolic and stress-responsive contexts. Overall, the IPA results suggest a predicted pathway-level convergence of the GP5-associated cmiRNAs on interconnected metabolic and signaling pathways. These findings are presented as pathway-level associations and should be interpreted as hypothesis-generating, without implying direct mechanistic relationships or causal inference (Figure 3 and Supplementary Table S3).

**Figure 4:**
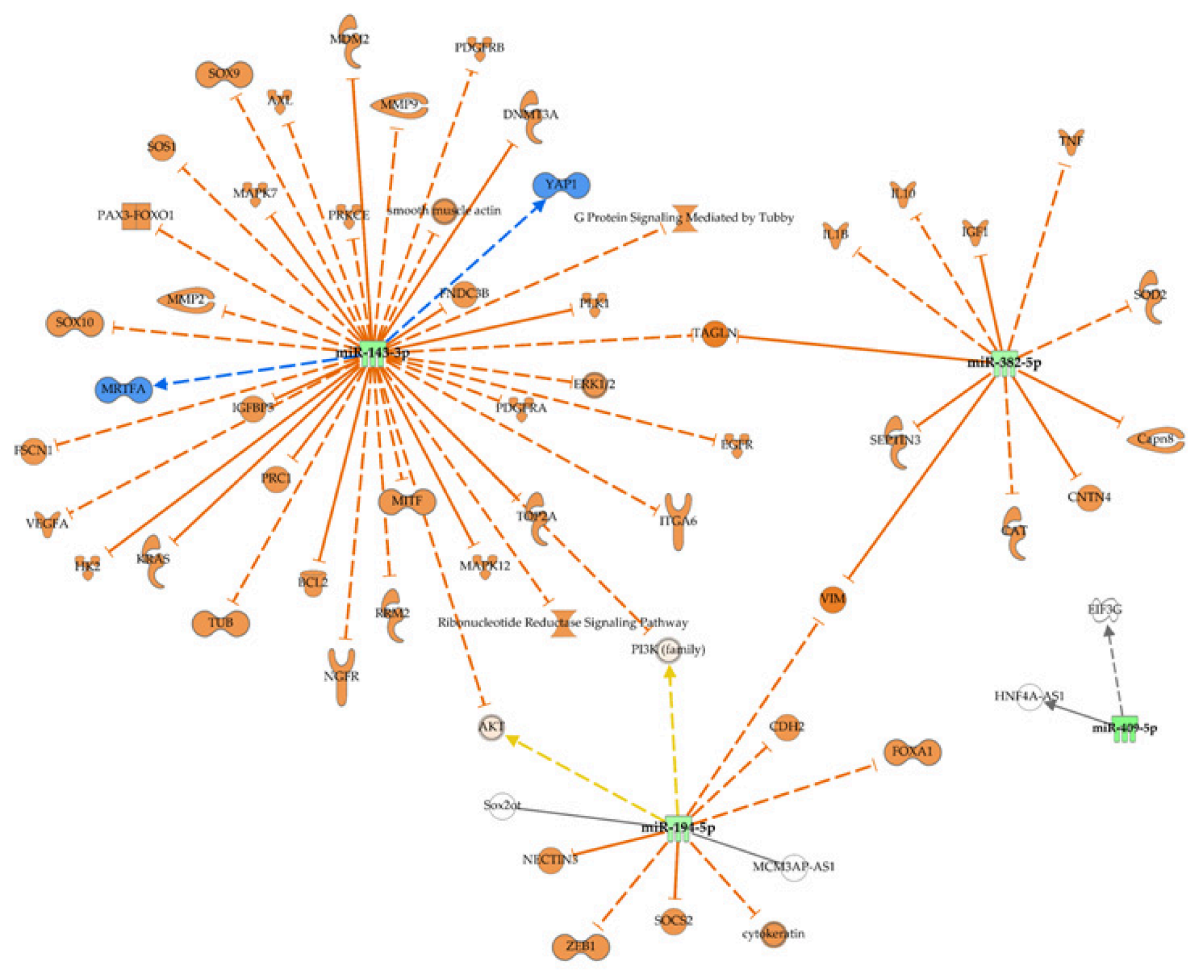
IPA–generated miRNA–target interaction network. Downregulated cmiRNAs (green nodes) are connected to predicted target genes through direct (solid lines) and indirect (dashed lines) interactions. Edge color indicates the predicted effect on downstream targets: activation (orange), inhibition (blue), inconsistent prediction (yellow), or no prediction (gray). The network illustrates the distribution of the GP5-modulated cmiRNAs within the reconstructed interaction landscape and their connections to shared regulatory nodes. This representation provides a visual overview of the inferred relationships underlying the pathway-level results and should be interpreted as a network-based integration of predicted interactions rather than direct mechanistic evidence.

To complement canonical pathway analysis, the IPA-generated miRNA–target interaction network was also examined as a visual representation of the inferred regulatory architecture. In this network, the GP5-modulated cmiRNAs converged on shared signaling nodes, providing a graphical overview of the degree of integration underlying the observed pathway-level associations (Figure 4).

## 4. Discussion

Thirty days of GP supplementation were associated with changes in the circulating miRNA profile of dairy ewes, characterized by a selective four-miRNA signature. Importantly, RT-qPCR analysis confirmed the absence of significant differences among CTRL T0, CTRL T30, and GP5 T0 samples (*p* > 0.05), indicating both temporal stability in the CTRL group and baseline comparability between CTRL and GP5 animals prior to supplementation. Moreover, although small RNA sequencing was performed only in the GP5 group, the subsequent RT-qPCR validation included both experimental groups and demonstrated significant temporal changes exclusively in GP5-fed animals. Notably, the animals included in the present study correspond to the same lactating cohort previously characterized by Guelfi et al. [8], in which clinical status, hematological profile, and biochemical parameters were comprehensively evaluated throughout the experimental period. As reported in that study, all animals remained clinically healthy, and hematological and biochemical variables remained within physiological reference ranges without evidence of time-dependent systemic alterations. Together, these independent observations support the interpretation that the validated cmiRNA changes are associated with GP supplementation rather than being explained solely by temporal physiological variation.

The limited number of differentially expressed molecules points to a focused post-transcriptional adjustment rather than a broad remodeling of the cmiRNA repertoire. The full concordance between limma-voom and DESeq2 further strengthens the robustness of this signature, confirming that the observed modulation is not dependent on the analytical framework. Overall, the coherent downregulation of miR-143, miR-409-3p, miR-382-5p, and miR-194 suggests a coordinated reduction in post-transcriptional regulatory constraint, rather than a generalized transcriptional perturbation, during exposure to GP bioactives.

The four-miRNA signature maps to physiological networks that are central to metabolic and inflammatory regulation in ruminants. Among them, miR-143 emerges as a key regulatory hub, acting as a conserved orchestrator of lipid handling, adipocyte differentiation, and glucose homeostasis, functions extensively documented across species [28]. Its expression in ruminant adipose and metabolically active tissues supports its involvement in nutrient sensing and metabolic regulation during dietary adaptation. miR-382-5p, although less explored in small ruminants, has been linked in other mammals to mitochondrial efficiency, oxidative balance, and cellular stress integration [29]. Fluctuations of this miRNA in circulation have been associated with shifts in metabolic demand, suggesting that its downregulation may reflect a controlled recalibration of mitochondrial and redox-responsive pathways, rather than a stress-related response, under polyphenol exposure. miR-409-3p participates in the control of inflammatory mediators through SOCS3/STAT3-dependent mechanisms [30], positioning it within regulatory axes that integrate immune activity and metabolic control—pathways known to respond dynamically to dietary bioactives in ruminants. miR-194, widely detectable in ruminant tissues and biofluids, including milk, has been associated with epithelial stability, nutrient-responsive signaling, and tissue homeostasis [31], supporting a systemic role in inter-organ metabolic communication during nutritional transitions.

Although both dietary groups received isoenergetic and isonitrogenous pelleted formulations, only GP5 animals exhibited a significant reduction in plasma glucose between T0 and T30, while CTRL ewes showed only minimal, non-significant fluctuations within their physiological range [8]. This divergence indicates that the decrease in circulating glucose is not attributable to dietary energy density per se, but rather reflects a diet-associated metabolic adjustment associated with grape pomace inclusion. GP is rich in insoluble fiber and tannin–proanthocyanidin complexes, which reduce the availability of rapidly fermentable carbohydrates and shift the volatile fatty acid pattern toward acetate at the expense of propionate, the major gluconeogenic precursor in ruminants [32,33]. A reduced propionate supply consequently limits hepatic gluconeogenesis, leading to lower circulating glucose, a profile fully consistent with the established role of propionate as the primary driver of hepatic gluconeogenesis in ruminants [34,35].

The coordinated downregulation of oar-miR-143, oar-miR-409-3p, oar-miR-382-5p, and oar-miR-194 aligns with this metabolic shift, as all four miRNAs regulate pathways involved in adipogenesis, gluconeogenic flux, lipid handling, mitochondrial function, and immune–metabolic integration [28,29,36,37]. Their concerted suppression therefore delineates a transition from lipid storage toward lipid utilization, representing a post-transcriptional signature consistent with a more energy-efficient metabolic state, rather than an indication of metabolic strain.

Specifically, 5% GP supplementation preserved systemic health without inducing alterations in hepatic, renal, lipid, or energy-related parameters, demonstrating that GP inclusion did not impose metabolic strain but rather supported a balanced physiological state [8]. The selective modulation of only four cmiRNAs is consistent with this phenotype: instead of signaling broad metabolic disruption, the cmiRNA response appears to reflect a controlled regulatory adjustment that parallels the mild shifts reported in productive and metabolic traits. Accordingly, the uniform downregulation of miR-143, miR-382-5p, miR-409-3p, and miR-194 supports the concept of nutritional fine-tuning, reinforcing the view that GP supplementation acts as a metabolic modulator rather than a metabolic stimulant.

This interpretation is further supported by the known biological activity of dietary polyphenols in ruminants. Polyphenols are increasingly recognized as modulators of cellular signaling in ruminants, acting through mechanisms that extend beyond their antioxidant properties. Several studies have shown that grape-derived polyphenols influence pathways related to oxidative balance, mitochondrial efficiency, and immunometabolic regulation in livestock species, often through subtle epigenetic mechanisms involving non-coding RNAs [1,4]. In dairy ewes, polyphenols can attenuate post-prandial oxidative stress, enhance ruminal fermentation efficiency, and modulate lipid and glucose utilization, supporting a systemic environment consistent with metabolic adaptation without inflammatory activation. These effects are compatible with the coordinated downregulation of miR-143, miR-382-5p, miR-409-3p, and miR-194 observed in the present study, as each of these cmiRNAs is linked to pathways influenced by polyphenolic metabolites-including AMPK activation, mitochondrial regulation, and immune–metabolic cross-talk. Emerging evidence across mammals suggests that dietary polyphenols can shape the cmiRNA pool by altering transcriptional activity, miRNA processing, and RNA stability. Such regulation is typically selective rather than global, producing focused signatures that mirror the nutrient-responsive pathways engaged during dietary adaptation [28]. The restricted four-miRNA pattern detected here is consistent with this paradigm, reinforcing the idea that GP supplementation is consistent with selective and potentially reversible epigenetic modulation rather than widespread disruption of post-transcriptional networks [15]. Overall, the available literature supports a model in which GP-derived polyphenols exert selective regulatory effects on both metabolic and inflammatory circuits, providing a coherent biological framework for the cmiRNA profile identified in this study.

In this context, IPA provided an integrative framework to interpret the biological coherence of the four-miRNA signature identified following GP5 supplementation. Rather than reflecting independent molecular events, the reconstructed IPA networks indicate that validated and high-confidence predicted targets of the modulated cmiRNAs converge on a limited number of shared regulatory hubs, as inferred from network topology and directionality, supporting the presence of a coordinated system-level adjustment [12]. A prominent convergence emerged on PPAR signaling, with PPARA and PPARG acting as heterodimers with RXRA and binding to nuclear PPRE elements, positioned as central nodes integrating pathways related to lipid handling, fatty acid oxidation, adipocyte differentiation, and broader energy metabolism. In this framework, the uniform downregulation of cmiRNAs such as miR-143 and miR-382-5p is consistent with a selective reduction in post-transcriptional constraint on metabolic regulators, as inferred from IPA-based target integration, rather than with a global transcriptional reprogramming, in line with the established role of miRNAs as fine-tuners of metabolic and adipose tissue homeostasis [38]. Importantly, the IPA suggested that the predicted attenuation of PPAR-dependent metabolic programs was accompanied by predicted activation of complementary signaling pathways within the reconstructed network. In particular, the cholecystokinin/gastrin-mediated signaling pathway was predicted by IPA to be activated, with regulatory axes converging on MAPK/ERK- and AP-1-dependent transcriptional programs and identifying PTGS2 (COX-2) as a key downstream node. Although gastrin and CCK are classically recognized as gastrointestinal hormones regulating digestive physiology, they have been shown to engage broad intracellular signaling networks with effects beyond acid secretion and enzyme release, including cell proliferation and MAPK-linked pathways [39].

In parallel, the predicted activation of the S100 family signaling pathway further supports this integrated view. Members of the S100 family, including S100A8, S100A9, and S100A12, serve as calcium-binding regulatory proteins implicated in immune and metabolic functions. Expression of these S100 calgranulin genes has been shown to be modulated in adipose tissue in response to dietary and inflammatory stimuli, with potential roles in adipocyte biology and immune cell recruitment [40]. Within the IPA framework, S100 family nodes connected adipocyte-related regulators such as PPARG and TP53 with immune-related hubs including NF-κB and MAPK pathways, delineating a network in which S100-dependent signaling is associated with adipogenesis, insulin sensitivity, and immune regulatory processes. Collectively, the IPA results suggest predicted pathway-level associations between the GP5-associated cmiRNA signature and interconnected metabolic and immune signaling networks, characterized by predicted attenuation of PPAR-driven metabolic programs alongside activation of CCK/gastrin- and S100-dependent pathways. Notably, while all four cmiRNAs were consistently downregulated at the expression level, IPA-based pathway reconstruction indicated that miR-143-3p and miR-382-5p contributed most prominently to the predicted pathway-level associations. In contrast, miR-409-3p and miR-194, despite being robustly modulated, showed a more dispersed target distribution and were primarily represented in the broader miRNA–target interaction network rather than in canonical pathway enrichment. This pattern suggests that these miRNAs may contribute to context-specific or tissue-related regulatory processes that are not fully captured by pathway-centric algorithms, reinforcing the view that the four-miRNA signature encompasses both core metabolic regulators and auxiliary modulators of dietary adaptation. The absence of enrichment for pathways associated with oxidative stress, cellular damage, or acute inflammation, together with the stability of systemic health parameters observed in the same animals, supports the interpretation that these network changes reflect physiological immune–metabolic integration rather than a pathological stress response. Overall, the IPA results provide hypothesis-generating evidence that the identified cmiRNAs may be associated with a selective post-transcriptional response to GP supplementation, consistent with the concept of epigenetic regulation as a dynamic interface between environmental cues and physiological phenotypes [41]. While IPA does not imply causality, the consistency between the reconstructed network, the cmiRNA profile, and the observed metabolic phenotype strengthens the biological plausibility of a nutritionally driven epigenetic adjustment [41]. The study presents some inherent constraints that should be considered when interpreting the findings. The sample size, although typical for controlled nutritional trials in ruminants, remains modest and may limit the detection of subtler effects on low-abundance cmiRNAs. The analysis focused exclusively on circulating small RNAs, without parallel quantification of mRNA targets in metabolically active tissues, preventing direct validation of the inferred regulatory relationships. In addition, no functional validation (e.g., target gene expression analysis, protein-level assessment, or in vitro modulation studies) was performed, limiting the ability to establish causal links between cmiRNA modulation and the predicted pathway-level effects. The lack of detailed information on pelleting conditions, including conditioning temperature, processing duration, pellet size, and moisture immediately after pelleting, represents a methodological limitation, as the potential effects of these parameters on the stability of GP bioactive compounds could not be assessed. Moreover, the 30-day duration captures an acute dietary response but does not allow evaluation of long-term stability or potential cumulative effects of grape pomace supplementation. In essence, the four-miRNA signature uncovered in this study emerges as a coherent molecular signature of dietary adaptation to GP supplementation, highlighting how dietary polyphenols can subtly recalibrate systemic regulation in dairy ruminants through targeted miRNA-mediated post-transcriptional mechanisms, while remaining consistent with a physiologically stable metabolic state, in line with the notion that epigenetic processes act as a dynamic interface between environment and physiology [41].

## 5. Conclusions

GP5 supplementation was associated with selective and reproducible changes in the cmiRNA profile of dairy sheep, characterized by a consistent four-miRNA signature comprising miR-143, miR-382-5p, miR-409-3p, and miR-194. This signature is consistent with a targeted post-transcriptional response involving cmiRNAs implicated in metabolic and immune–metabolic regulation. Integration of miRNA profiling with hypothesis-generating pathway analysis indicated predicted attenuation of PPAR-related metabolic signaling, together with predicted activation of cholecystokinin/gastrin- and S100-related pathways. These pathway-level associations were observed in the absence of enrichment for pathways related to oxidative stress, cellular damage, or acute inflammation and were consistent with the previously demonstrated metabolic safety and stability of systemic health parameters in the same animal cohort. Overall, these findings support the biological relevance of the identified four-miRNA signature and suggest that GP supplementation may be associated with a selective regulatory adjustment rather than a generalized metabolic perturbation.

## Figures and Tables

**Table 1:** List of miRCURY LNA™ microRNA PCR assays used for spike-in quality controls, ER selection, and qPCR validation of differentially expressed (DE) cmiRNAs. UniSp2 and UniSp4 were used as RNA isolation spike-ins, whereas UniSp6 served as the reverse transcription (RT) spike-in control. Three cmiRNAs (oar-miR-191-5p, oar-miR-16b-5p, oar-miR-103a-3p) were selected as ERs based on their expression stability. Four assays targeted the cmiRNAs identified as differentially expressed in GP5 animals by NGS analysis (oar-miR-143, oar-miR-382-5p, oar-miR-409-3p, and oar-miR-194). For some targets, species-specific ovine assays were not available; therefore, miRCURY LNA™ assays originally designed for other species were used. According to the manufacturer, these assays target conserved miRNA sequences and are validated to cross-react with the corresponding ovine miRNAs. Abbreviations: oar, *Ovis aries* (sheep); cfa, *Canis familiaris* (dog); hsa, *Homo sapiens* (human).

Primer	Recommended for	GeneGlobe ID
UniSp2	Spike-in (to assess RNA isolation efficiency)	YP00203950
UniSp4	Spike-in (to assess RNA isolation efficiency)	YP00203953
UniSp6	Spike-in (assessment of RT efficiency)	YP00203954
cfa-miR-191	ER cmiRNA	YP00205972
oar-miR-16b	ER cmiRNA	YP02115941
oar-miR-103	ER cmiRNA	YP02110471
hsa-miR-143-3p	Target DE cmiRNA	YP00205992
oar-miR-409-3p	Target DE cmiRNA	YP02109129
hsa-miR-382-5p	Target DE cmiRNA	YP00204169
hsa-miR-194-5p	Target DE cmiRNA	YP00204080

**Table 2:** For each cmiRNA, the table reports log_2_ fold change (logFC), linear fold-change, average log_2_ expression (AveExpr), *p*-values, and FDR-adjusted significance. All four cmiRNAs showed significant downregulation (FDR < 0.05), with oar-miR-409-3p displaying the greatest reduction at T30.

cmiRNA	log_2_FC	1/(2^(logFC))	Ave Expr	*p* Value	FDR (adj.*p*.Val)	Trend
miR-409-3p	−1.32	2.50	6.706	0.000944	0.0359	↓ GP5 T30
miR-143-3p	−1.14	2.20	11.499	0.000721	0.0359	↓ GP5 T30
miR-194-5p	−0.94	1.91	6.299	0.00027	0.0322	↓ GP5 T30
miR-382-5p	−0.92	1.90	8.511	0.000423	0.0322	↓ GP5 T30

**Table 3:** Stability ranking of candidate endogenous reference cmiRNAs across all experimental conditions. Expression stability was evaluated using RT-qPCR Cq data from CTRL and GP5 samples at T0 and T30 through the ΔCt method, BestKeeper, NormFinder, and geNorm, together with the comprehensive RefFinder ranking. For each method, candidates are ordered from most stable to least stable. Oar-miR-16b-5p was identified as the most stable individual candidate, followed by cfa-miR-191-5p, whereas oar-miR-103a-3p showed the lowest stability.

Algorithm	Most Stable	Moderately Stable	Least Stable
ΔCt method	oar-miR-16b	cfa-miR-191	oar-miR-103
BestKeeper	oar-miR-16b	cfa-miR-191	oar-miR-103
NormFinder	oar-miR-16b	cfa-miR-191	oar-miR-103
GeNorm	cfa-miR-191/oar-miR-16b		oar-miR-103
RefFinder	oar-miR-16b	cfa-miR-191	oar-miR-103

**Table 4:** Two-way repeated-measures ANOVA of RT-qPCR data (2^−ΔCq values). Group (CTRL vs. GP5) and Time (T0 vs. T30) were included as fixed factors. F-values, degrees of freedom [F(1,10)], and corresponding *p*-values are reported for the Group effect, Time effect, and Group × Time interaction. Planned pairwise comparisons were subsequently performed using Bonferroni’s multiple-comparison test. ns, not significant.

MiRNA	Group Effect F(1,10), *p*	Time Effect F(1,10), *p*	Group × Time Interaction F(1,10), *p*	CTRL T0 vs. T30	GP5 T0 vs. T30
miR-143	34.08 (0.0002)	51.71 (<0.0001)	51.71 (<0.0001)	ns	*p* < 0.001
miR-409	27.50 (0.0004)	48.53 (<0.0001)	48.53 (<0.0001)	ns	*p* < 0.001
miR-194	21.75 (0.0009)	59.57 (<0.0001)	59.57 (<0.0001)	ns	*p* < 0.001
miR-382	9.06 (0.0131)	6.52 (0.0287)	6.52 (0.0287)	ns	*p* < 0.01

## Data Availability

The original contributions presented in this study are included in the article/Supplementary Materials. Further inquiries can be directed to the corresponding authors.

## Supplementary Materials

The following supporting information can be downloaded at: https://www.mdpi.com/article/10.3390/ani16152305/s1, Figure S1. UHPLC–HRMS chromatogram of grape pomace extract; Table S1. UHPLC–HRMS identification of grape pomace compounds; Table S2a. Composition of the experimental diets; Table S2b. Proximate composition of the experimental diets; Table S3. IPA signaling network components and functional annotations. Supplementary NGS data.xlsx.
