# Supplementary material for: Serum miRNA Changes Associated with Grape Pomace Supplementation in Dairy Sheep
Source: Animals (Basel). 2026 Jul 25;16(15):2305. doi: 10.3390/ani16152305 (PMC13463471; doi:10.3390/ani16152305)
Supplement: Supplementary file 1 [file animals-16-02305-s001.zip › Supplementary materials.pdf]

Supplementary Table S1. UHPLC–HRMS identification of grape pomace compounds.

Identified compounds in grape pomace extract by UHPLC–HRMS. Peaks correspond to those detected in the base peak chromatogram, with molecular formula, exact mass, and fragmentation profile. The notation nf indicates cases where no characteristic MS/MS fragments were detected or fragmentation data were not available.

Pk. no.	RT (min)	[M-H]⁻ *m/z* Calculated	[M-H]⁻ *m/z* Observed	Predicted Molecular Formula	MS/MS *m/z*	Compound Identity
**1**	1.81	191.0561	191.0568	$C_7H_{12}O_6$	85 (100)	Quinic acid
**2**	1.92	133.0143	133.0144	$C_4H_6O_5$	115 (100)	Malic acid
**3**	1.95	149.0081	149.0092	$C_4H_6O_6$	103, 87 (100), 72, 59	Tartaric acid
**4**	3.21	117.0182	117.0194	$C_4H_6O_4$	99, 73 (100)	
**5**	8.09	331.066	331.067	$C_{13}H_{16}O_{10}$	169 (100)	
**6**	8.86	577.1352	577.1351	$C_{30}H_{26}O_{12}$	407, 289 (100), 245, 203, 201, 161, 125	Procyanidin B2
**7**	9.63	289.0718	289.0716	$C_{15}H_{14}O_6$	245, 203, 151, 125, 123, 109 (100)	+/- Catechin
**8**	9.96	577.1352	577.1351	$C_{30}H_{26}O_{12}$	407, 289 (100), 245, 203, 201, 161, 125	Procyanidin like
**9**	10.52	729.145	729.1459	$C_{30}H_{34}O_{21}$	407 (100), 289, 169, 125	
**10**	10.9	289.0718	289.0716	$C_{15}H_{14}O_6$	245, 203, 151, 125, 123, 109 (100)	Epicatechin
**11**	11.83	197.0445	197.0455	$C_9H_{10}O_5$	nf	
**12**	12.09	441.0816	441.0826	$C_{22}H_{18}O_{10}$	203 (100), 201, 169, 125	
**13**	12.48	577.1352	577.1355	$C_{30}H_{26}O_{12}$	407, 289 (100), 245, 203, 201, 161, 125	Procyanidin like
**14**	12.72	509.129	509.1302	$C_{23}H_{26}O_{13}$	nf	
**15**	13.73	363.0711	363.072	$C_{17}H_{16}O_9$	nf	
**16**	14.66	655.1658	655.1669	$C_{25}H_{36}O_{20}$	nf	
**17**	14.66	463.0882	463.0882	$C_{21}H_{21}O_{12}$	300 (100)	Quercetin 3-*β*-d-glucoside
**18**	15.7	477.0664	477.0675	$C_{21}H_{18}O_{13}$	nf	
**19**	16.32	271.0612	271.0611	$C_{15}H_{12}O_5$	151 (100)	Naringenin
**20**	17.36	301.0354	301.0352	$C_{15}H_{10}O_7$	203, 178, 151 (100), 121	Quercetin
**21**	18.58	613.343	613.3442	$C_{28}H_{54}O_{14}$	493 (100), 475, 331, 2023	
**22**	18.88	285.0405	285.0404	$C_{15}H_9O_6$	203, 151 (100), 107	Luteolin
**23**	20.54	207.138	207.139	$C_{13}H_{20}O_2$	152, 135, 133, 121, 101 (100), 55	
**24**	21.37	313.0707	313.0717	$C_{17}H_{14}O_6$	225, 203, 181, 121 (100)	
**25**	23.48	383.0761	383.0769	$C_{20}H_{16}O_8$	339, 311, 225, 203, 191, 121 (100)	
**26**	26.38	471.3479	471.348	$C_{30}H_{48}O_4$	203 (100)	Maslinic acid
**27**	27.97	367.2632	367.2639	$C_{25}H_{36}O_2$	273, 225, 203, 177 (100)	
**28**	28.06	455.3531	455.3529	$C_{30}H_{47}O_3$	203 (100)	Ursolic acid
**29**	28.21	279.2319	279.2328	$C_{18}H_{32}O_2$	261, 225, 203 (100), 176, 148	

Base peak chromatogram of grape pomace extract analyzed by UHPLC–HRMS in negative ionization mode. Peaks 1–29 correspond to the polyphenolic compounds identified and listed in Table S1.

Supplementary Figure S1. UHPLC–HRMS chromatogram of grape pomace extract.

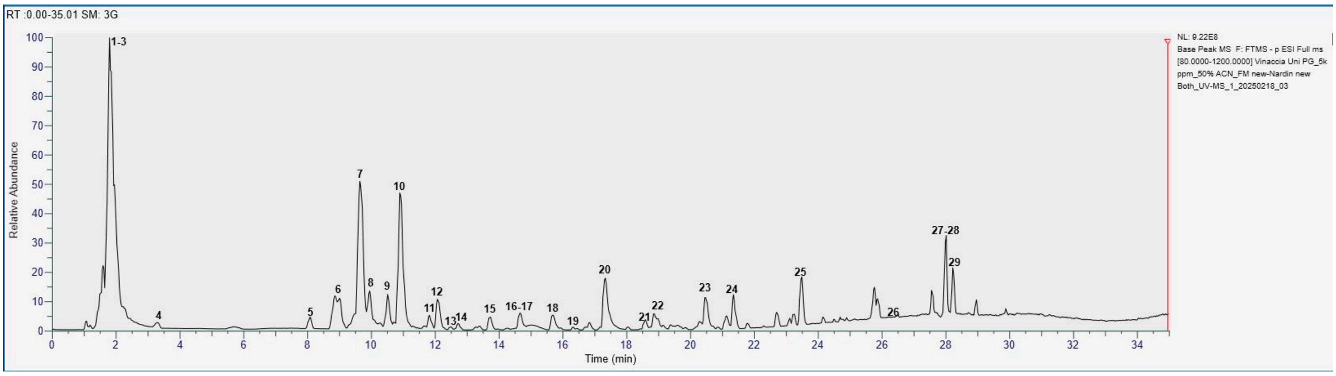

Supplementary Table S2a. Composition of the experimental diets.

Values are expressed as percentage of the final pelleted diet (as-fed basis). The GP5 diet was formulated by replacing 5% of the CTRL diet with dried grape pomace.

Ingredient	CTRL	GP5
Corn	27.80	27.00
Wheat bran	27.00	25.00
Durum wheat middlings	26.50	24.30
Soybean meal	5.30	5.30
Maize germ meal	5.00	5.00
Molasses concentrate	2.50	2.50
Calcium carbonate	2.00	2.00
Sugar cane molasses	2.00	2.00
Sodium bicarbonate	0.80	0.80
Sodium chloride	0.50	0.50
Urea	0.30	0.30
Sheep vitamin–mineral premix	0.30	0.30
**Dried grape pomace**	**0.00**	**5.00**

Supplementary Table S2b. Proximate composition of the experimental diets.

Proximate chemical composition of the CTRL and GP5 pelleted diets (% as-fed basis). Analytical values refer to the final pelleted diets.

Analytical composition (% as-fed basis)	CTRL	GP5
Dry matter	86.78	86.92
Crude protein	15.43	15.24
Ether extract	2.85	2.90

Crude fiber	5.65	7.02
Lignin	3.58	3.93
Ash	6.74	6.70
Calcium	0.86	0.89
Total phosphorus	0.51	0.48
Sodium	0.50	0.51

Supplementary Table S3. IPA signaling network components and functional annotations.

The table comprehensively reports all genes, molecular nodes, and functional outputs explicitly represented in the IPA-derived PPAR, S100, and cholecystokinin/gastrin-mediated signaling networks shown in Figure 3. For each node, the corresponding full name and KEGG-annotated biological function are provided to support functional interpretation in the context of metabolic and immune regulation. KEGG pathway annotations refer to the human KEGG database and were used solely for functional classification, in accordance with the cross-species gene mapping implemented in IPA.

	Gene / Node	Full name	KEGG pathway ID(s)	KEGG-annotated biological function
**PPAR**	**INSULIN**	Insulin	hsa04910 (Insulin signaling)	Hormonal regulation of glucose uptake and systemic metabolic homeostasis
	**INSR**	Insulin Receptor	hsa04910 (Insulin signaling)	Initiation of insulin-mediated metabolic signaling
	**PDGF**	Platelet-Derived Growth Factor	hsa04010 (MAPK signaling)	Growth factor activating receptor-mediated MAPK signaling
	**PDGFR**	Platelet-Derived Growth Factor Receptor	hsa04010 (MAPK signaling)	Receptor tyrosine kinase triggering MAPK cascade
	**MAP2K1/2**	Mitogen-Activated Protein Kinase Kinase 1/2	hsa04010 (MAPK signaling)	Activation of ERK1/2 downstream of receptor signaling
	**MAPK1/MAPK3 (ERK1/2)**	Extracellular Signal-Regulated Kinases 1/2	hsa04010 (MAPK signaling)	Integration of growth factor and metabolic signals
	**NR0B2 (SHP)**	Nuclear Receptor Subfamily 0 Group B Member 2	hsa03320 (PPAR signaling)	Transcriptional corepressor modulating nuclear receptor activity
	**NCOA**	Nuclear Receptor Coactivator	hsa04919; hsa04915	Transcriptional coactivation of nuclear receptor targets
	**NCOR**	Nuclear Receptor Corepressor	hsa04919; hsa04350	Repression of nuclear receptor–mediated transcription
	**RXRA**	Retinoid X Receptor Alpha	hsa03320 (PPAR signaling)	Heterodimeric partner enabling PPAR-dependent transcription
	**PPARA**	Peroxisome Proliferator-Activated Receptor Alpha	hsa03320 (PPAR signaling)	Regulation of fatty acid uptake and β-oxidation
	**PPARG**	Peroxisome Proliferator-Activated Receptor Gamma	hsa03320 (PPAR signaling)	Control of adipogenesis and insulin sensitivity
	**PPRE**	Peroxisome Proliferator Response Element	Cis-regulatory DNA element	DNA element mediating PPAR/RXR transcriptional activity
	**Peroxisome proliferation**	Functional output	hsa03320 (PPAR signaling)	Expansion of peroxisomal compartment
	**Fatty acid oxidation**	Functional output	hsa03320 (PPAR signaling)	β-oxidation of fatty acids
	**Fatty acid degradation**	Functional output	hsa03320 (PPAR signaling)	Catabolic processing of fatty acids

	**Lipid homeostasis**	Functional output	hsa03320 (PPAR signaling)	Maintenance of systemic lipid balance
	**Adipocyte differentiation**	Functional output	hsa03320 (PPAR signaling)	Regulation of adipocyte maturation
	**Macrophage function**	Functional output	hsa03320 (PPAR signaling)	Modulation of macrophage lipid handling
	**Glucose uptake**	Functional output	hsa04910 (Insulin signaling)	Cellular glucose uptake in response to insulin
**S100**	**S100A16**	S100 Calcium-Binding Protein A16	hsa04064; hsa04657	Regulation of adipocyte function and metabolic stress responses
	**TP53**	Tumor Protein P53	hsa04115 (p53 signaling)	Regulation of cell cycle control and inhibition of adipogenesis
	**Insulin resistance**	Functional output	hsa04931 (Insulin resistance)	Impaired insulin signaling in adipocytes
	**Proliferation of adipocytes**	Functional output	hsa04110 (Cell cycle)	Regulation of adipocyte precursor proliferation
	**Adipogenesis**	Functional output	hsa03320 (PPAR signaling)	Differentiation of preadipocytes into adipocytes
	**Insulin sensitivity**	Functional output	hsa04910 (Insulin signaling)	Maintenance of insulin responsiveness
**Cholecystokinin Gastrin**	**GH1**	Growth Hormone 1	hsa04935 (Growth hormone signaling)	Endocrine modulation of metabolic and growth-related pathways
	**IL1**	Interleukin-1	hsa04657 (IL-17 signaling)	Pro-inflammatory cytokine influencing hormonal signaling
	**Gastric acid**	Gastric acid secretion	hsa04972 (Gastric acid secretion)	Effector of gastrin-mediated digestive function
	**SST**	Somatostatin	hsa04972 (Gastric acid secretion)	Inhibitory hormone regulating gastrin release
	**GAST**	Gastrin	hsa04972 (Gastric acid secretion)	Hormonal regulator of gastric acid secretion
	**CCK**	Cholecystokinin	hsa04972 (Gastric acid secretion)	Digestive hormone activating GPCR signaling
	**CCKBR**	Cholecystokinin B Receptor	hsa04972 (Gastric acid secretion)	GPCR mediating gastrin/CCK signaling
	**EGFR**	Epidermal Growth Factor Receptor	hsa04012 (ErbB signaling)	Receptor tyrosine kinase activating MAPK signaling
	**GNAQ**	G Protein Subunit Alpha Q	hsa04020 (Calcium signaling)	GPCR signal transduction to PLC
	**PLCB**	Phospholipase C Beta	hsa04020 (Calcium signaling)	Generation of IP3 and DAG
	**PIP2**	Phosphatidylinositol 4,5-bisphosphate	hsa04070 (Phosphatidylinositol signaling)	Substrate for second messenger generation
	**IP3**	Inositol 1,4,5-trisphosphate	hsa04020 (Calcium signaling)	Calcium mobilization
	**DAG**	Diacylglycerol	hsa04020 (Calcium signaling)	Activation of PKC
	**PKC**	Protein Kinase C	hsa04010 (MAPK signaling)	Link between GPCR and MAPK cascades
	**MAP2K5**	Mitogen-Activated Protein Kinase Kinase 5	hsa04010 (MAPK signaling)	Activation of ERK5
	**MAPK7**	Mitogen-Activated Protein Kinase 7 (ERK5)	hsa04010 (MAPK signaling)	Hormone-responsive transcriptional regulation
	**MAP3K6**	Mitogen-Activated Protein Kinase Kinase Kinase 6	hsa04010 (MAPK signaling)	Stress-activated MAPK signaling
	**MAPK14**	Mitogen-Activated Protein Kinase 14 (p38α)	hsa04010 (MAPK signaling)	Integration of inflammatory and metabolic signals
	**EPHA4**	Ephrin Type-A Receptor 4	hsa04360 (Axon guidance)	Modulation of MAPK and cellular signaling

**ICER**	Inducible cAMP Early Repressor	hsa04024 (cAMP signaling)	Transcriptional repression downstream of GPCR signaling
**JNK**	c-Jun N-terminal Kinase	hsa04010 (MAPK signaling)	Regulation of AP-1 activity
**JUN**	Jun Proto-Oncogene	hsa04010 (MAPK signaling)	AP-1 transcription factor component
**FOS**	Fos Proto-Oncogene	hsa04010 (MAPK signaling)	AP-1 transcription factor component
**ATF2**	Activating Transcription Factor 2	hsa04010 (MAPK signaling)	MAPK-responsive transcription factor
**ELK1**	ETS Like-1 Protein	hsa04010 (MAPK signaling)	ERK-dependent transcriptional activation
**SRF**	Serum Response Factor	hsa04010 (MAPK signaling)	Regulation of immediate-early gene transcription
**MEF2**	Myocyte Enhancer Factor 2	hsa04010 (MAPK signaling)	Transcriptional integration of MAPK signals
**PTK2B**	Protein Tyrosine Kinase 2 Beta	hsa04010 (MAPK signaling)	Signal integration downstream of PKC
**PTGS2 (COX-2)**	Prostaglandin-Endoperoxide Synthase 2	hsa04068; hsa04064; hsa04010	Convergence node integrating hormonal, inflammatory, and MAPK signaling
